# Intervention and the Child With FAS

**Published:** 1994

**Authors:** Lyn Weiner, Barbara A. Morse

**Affiliations:** Lyn Weiner, M.P.H., is associate professor of psychiatry (public health), and Barbara A. Morse, Ph.D., is assistant research professor of psychiatry (psychology), at Boston University School of Medicine, Fetal Alcohol Education Program, Brookline, Massachusetts

## Abstract

Although the research is sparse, it appears that specific interventions may help children with FAS better overcome their cognitive and behavioral problems. Special strategies such as consistency, a structured environment, and attention to learning skills can help these children develop to their highest potential.

Fetal alcohol syndrome (FAS) affects children in several ways, with a considerable amount of variability in the levels of behavioral, psychological, and cognitive deficits (Rosett and Weiner 1984; Weiner and Morse 1991). Some children have only subtle problems, whereas others are so severely affected that, as they age, they cannot function in the community. People working with these children have tried to develop caretaking and teaching techniques that circumvent the disabilities. The goal of such intervention strategies is to afford all people affected by FAS every opportunity to thrive in society and to reach their maximum potential.

In the field of child development, there is a general view that early intervention and a facilitating environment can help to maximize every child’s potential, no matter what the problem. However, children without learning disabilities or behavioral problems can thrive without such interventions, whereas children such as those with FAS need interventions to approach normalcy.

## Understanding the Challenges of FAS

Before adequate interventions can be designed, the challenges of rearing and educating the diverse population of children with FAS must be understood. IQ’s of children with FAS range from 45 to 110,^1^ and the majority of affected children have mild to moderate behavior problems. However, the literature usually describes only the most critically affected children, who have IQ’s below 50 and have severe behavior problems. As a result, negative outcomes for all children with FAS have been predicted; they are often described as mentally retarded, lacking conscience and judgment, and prone to criminal activity. Such dire predictions fail to consider the enormous heterogeneity of the affected population and the potential benefits of targeted intervention strategies.

### CNS Problems of Children With FAS

Abnormalities of the brain and central nervous system (CNS) in children with FAS can create problems, including diminished intelligence, learning disabilities, inappropriate or unusual behaviors, delays in speech and language development, poor eating and sleeping patterns, and delayed developmental milestones (Rosett and Weiner 1984). Many of these problems are evident even in children with FAS who have normal intelligence. Studies that examine and describe the developmental problems of children with FAS provide some understanding of the special needs of these children that can lead to intervention strategies.

#### Research Problems

Studies conducted to date have some methodological problems. Few large, well-controlled studies have compared the progress of children with FAS with the progress of children with other developmental problems or with no problems. Instead, most studies measure the children’s intelligence, motor coordination, and attention span and compare the results with societal norms. The studies reviewed below include children who were tested only once or twice over time and who were not provided with specific or consistent interventions. Most were case studies of individual children who had been referred to research centers after receiving a diagnosis of FAS from their primary care providers, so they were unlikely to be a representative sample of children with FAS. The age at which the children were evaluated varied from infancy to adolescence. Few of these studies employed a control population.

#### CNS Problems

In a group of 20 children with FAS ranging in age from 9 months to 21 years, Streissguth and colleagues (1978) found that intelligence scores remained stable 1 to 4 years after the first testing, suggesting that the abilities of children with FAS do not improve over time. Similarly, Dehaene and colleagues (1977) reported that among a group of 16 children with FAS, IQ scores did not change over time for the most severely affected. In their study, Spohr and colleagues (1993) reported that psychiatric and learning problems persisted in children with FAS over time.

Aronson and Olegard (1987) observed that the development of children with FAS appeared normal at some times and delayed at other times. The children’s hand and eye coordination and their ability to perceive shapes and understand concepts were impaired. The majority of the children had severely disturbed visual perception or auditory perception or both. Their learning was further complicated by hyper-activity. Kodituwakku and colleagues (1992) reported that children with FAS (mean age 13 years) had significant deficits in flexible problem solving, regulating behavior, and planning and utilizing feedback. Don and colleagues (1993) found that a group of young adults with FAS (mean age 19 years) and IQ’s greater than 86 learned more slowly but retained the information well once they had learned it. They had difficulty with tasks requiring mental control and efficient processing. Traditional academic and intellectual testing did not fully reflect the cognitive disabilities of this group.

Some of the problems children with FAS face may result from exaggerated responses to their surroundings, known as sensory hypersensitivity (Wilbarger and Wilbarger 1991). Such responses suggest that the brain is unable to sort incoming sensory information effectively and consistently. Sensory hypersensitivity contributes to problems with eating, sleeping, activity levels, learning, and behavior. It also may cause others to incorrectly perceive children with FAS as being aggressive; for example, children with FAS may strike out at someone who has only gently bumped them.

### Can Interventions Help?

The studies of children with FAS who, in general, were not exposed to any kind of intervention strategy, found that the children’s symptoms remained constant over time (Streissguth et al. 1978; Dehaene et al. 1977). However, Spohr and colleagues (1993) observed improvements in symptoms in some children with FAS and associated these improvements with a supportive home environment. Aronson and Olegard (1987) also noted that a stable environment resulted in fewer psychosocial problems for children with FAS.

The association between environment and outcome suggests that children with FAS may learn to adapt to cognitive and behavioral problems and that early intervention may help maximize their potential. This hypothesis is supported by a few pilot programs.^2^ For example, Bierich (1978) observed that learning ability and other mental functions improved in moderately affected children when long-term occupational therapy, directed at small-muscle groups, reduced their hyperexcitability. Koranyi and Csiky (1978) reported that early medical and educational intervention alleviated some symptoms among five children with FAS (ages 2 to 13 years), although they did not reach normalcy. These researchers concluded that symptoms of FAS were influenced by changes in socioeconomic conditions, maternal-infant interactions, state of health, and timeliness of interventions.

In 1984, Zaleski (unpublished data) reported on an early intervention program in which one group of 14 preschool children with FAS was provided intensive early intervention,^3^ while another group received support but was not involved in any specific program. The intervention program in part focused on teaching parents techniques to stimulate their children at home. Zaleski reported that when they were retested after 6 months, children in the intensive program showed significant gains in intelligence scores compared with the children who were not in the program (actual scores were not reported).

Although there is a need for additional research, the pilot studies suggest that the nature of disability in children with FAS is complex and may differ from that found in children with developmental delays associated with factors other than fetal alcohol exposure, including fetal exposure to illicit drugs. Each child with FAS is unique, exhibiting some but not all of the behavioral characteristics; the children are alike, however, in that they all show some learning difficulties and behavior problems (Burgess and Streissguth 1992). Given the nature of the problems seen in children with FAS, specific interventions, targeted to their needs, should be the most effective strategies.

## FAS Intervention Strategies

### Sources of Interventions

To date, there have been no systematic investigations of effective interventions for FAS. Parent reports (from hot-line calls, newsletters, and support groups) and chapters in *Fantastic Antone Succeeds!*, a recently published book on teachers’ and parents’ experiences educating children with FAS (Kleinfeld and Wescott 1993), are currently the best sources for information about FAS intervention, although they are not derived from controlled research studies. Developed independently by parents and teachers, the interventions discussed below are based on concepts consistent with the research findings discussed above. The strategies focus primarily on CNS defects that affect intelligence and behavior. Parents and teachers who have used these intervention techniques have seen positive change in children whom no one thought could improve (Kleinfeld and Wescott 1993).

### Strategies

#### Environment

Restructuring the child’s environment is the primary intervention technique consistently described by parents, teachers, and others who have observed children with FAS. The goal of this strategy is to remove barriers to the child’s progress. If, for example, a child’s fine motor skills make writing laborious, this principle suggests providing the child with a typewriter or computer to remove the hurdle of writing by hand, allowing the child to move ahead. Tanner-Halverson (1993) applies this principle to the classroom, although it also can be used in other settings. She suggests organizing the classroom environment by defining specific work and play areas. Teachers should keep work spaces clear and free of distraction and put materials not currently used out of sight.

**Figure:**
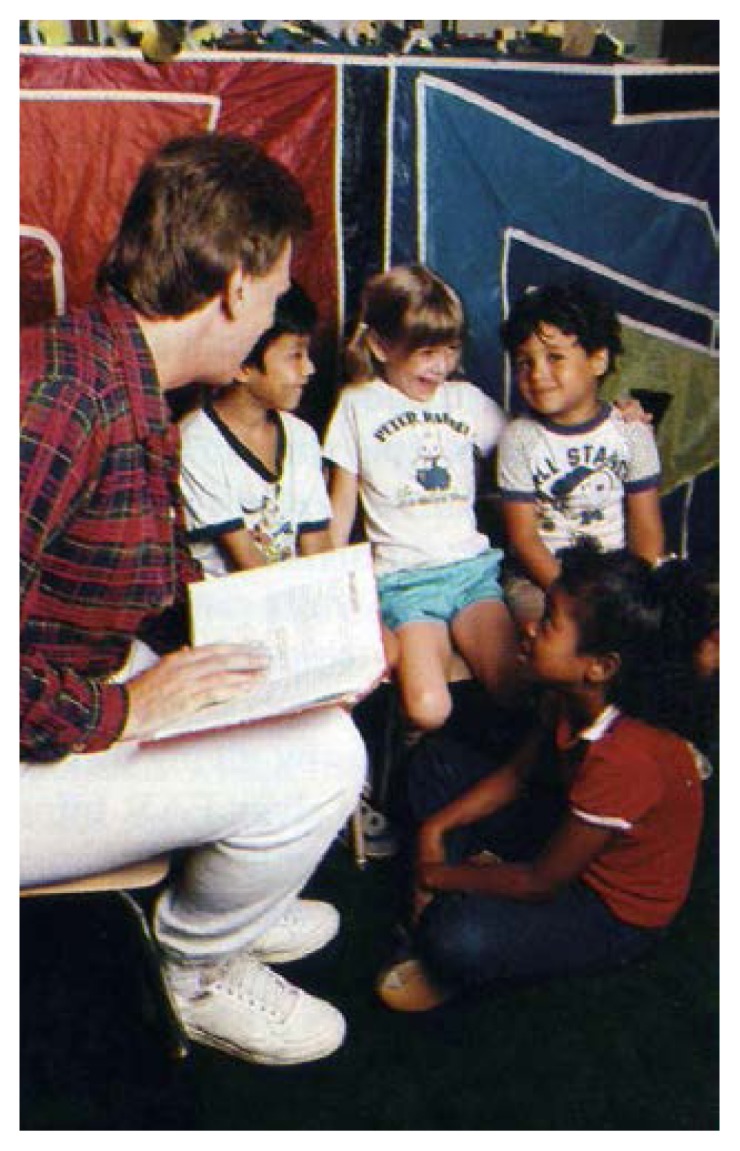
Children with FAS do best in context-specific learning environments in which the teacher uses visual and activity-based methods and creative, flexible strategies.

Murphy (1993) emphasizes the need to regulate sensory stimulation in the classroom, including touch, sound, and light. Because children with FAS are prone to sensory hypersensitivity, they may be easily overwhelmed in situations where they must process multiple stimuli at one time. Such hypersensitivity can severely and negatively impact their learning, attention, and behavior. Murphy recommends planning teaching activities that prevent mental or physical overstimulation, such as ensuring that reading occurs in a quiet room. According to Murphy, therapy to reduce hypersensitivity (i.e., sensory integration therapy, which desensitizes the child to touch, sound, and light) should be initiated as soon after birth as possible.

#### Learning Skills

A second intervention strategy is teaching FAS children how to learn. Children with FAS appear to learn and use information differently than does the average child (Kodituwakku et al. 1992). They must be carefully taught even the most basic skills, such as distinguishing friends from strangers. Winick (1993) has demonstrated that role playing can be effective in teaching children with FAS how to understand consequences and appropriate behavior. Phillpot and Harrison (1993) suggest that the emphasis should be on helping children find strategies of their own to improve memory and learning.

#### Consistency

A third intervention strategy is consistency in the environment, including the behavior and responses of people in the child’s world. Caldwell (1993) followed the experiences of her son, who has FAS, through four educational programs and found that he thrived and behaved appropriately in the classrooms that were most consistent. Although this is only the experience of one child, the programs that were successful for Caldwell’s son included:

- Teachers who were explicit, consistent, and used preventive discipline.
- Visual aids and class arrangement that reinforced class rules and activities.
- Program routines that varied little from day to day.
- Techniques that were designed to empower, not intimidate, children.

Hinde (1993) suggests that parents also should structure their children’s home environment. In her experience, the two most important factors for success in working with FAS children, birth to age 3, are the parents’ willingness to alter their lifestyles and make changes that allow their child to succeed, and the parents’ ability to provide a positive atmosphere, working from strengths that they have identified in their child. As a way of achieving success, Hinde teaches task analysis—breaking down each desired behavior into small steps and rewarding the child once each step is learned.

## Effective Interventions for Specific FAS Problems

Observations made over the last 20 years by researchers, clinicians, parents, and teachers have led to the informal development of interventions for specific problems that children with FAS encountered at home, school, and in the community (Morse and Weiner 1993). Some children with FAS are more severely affected than others, and none will have all of the problems discussed below. However, as a group, children with FAS display more developmental and behavioral problems than do children without FAS (Burgess and Streissguth 1992). The general intervention strategies of structuring the environment, teaching learning skills, maintaining consistency, and acknowledging the role of the sensory system as discussed above will help the child cope with these problems. Although severely affected children may experience only slight improvement, the majority will benefit to some degree or another.

### Sensory Hypersensitivity

Interventions to minimize sensory overload include diminishing intrusive sensations by providing loose clothing, supplying sunglasses to reduce glare, and avoiding crowded situations. When a child’s sensory system has been overloaded, the child may begin to repeat words and thoughts, shut down, throw temper tantrums, or exhibit frustration. Once this overload has occurred, it is important to reduce all stimulation for a period of time or to help a child use a calming technique—sitting in a beanbag chair, rocker, or hammock; taking a warm bath or shower; or listening to quiet music through headphones.

### Learning Difficulties

Difficulties with learning may be associated with organizational and processing deficits in the areas of information input, output, integration, and memory. When children with FAS have difficulties remembering and retrieving information, they may be perceived as willful or lazy. In addition, their learning often occurs in spurts, with apparently easy periods followed by difficult ones.

Children with FAS require context-specific learning environments; visual and activity-based methods; and creative, flexible strategies. They have difficulty applying information learned in one situation to another one. They often miss cues, such as gestures, facial expressions, and tone of voice, that help most people identify appropriate behavior. Visual information such as pictures and charts should back up all spoken information. The teacher’s use of a microphone while the child listens through headphones may improve the child’s focus and concentration.

### Inappropriate Social Skills

Some children with FAS often exhibit socially inappropriate behavior and are unable to consider the consequences of their actions. They may blur the distinctions between public and private behavior. Although these actions may be perceived as behavior problems, they actually reflect a learning disability.

Social skills may be improved through careful, repetitive, and concrete teaching of appropriate behavior. Also, clear, consistent, and immediate rewards and punishments help to reinforce positive behaviors and discourage negative ones, if they are clearly linked to the desired behaviors. For example, children who throw food should help clean the mess. Changes in routine and transitions that may trigger a child to display antisocial behavior should be explained well in advance, with role playing or practice sessions used to ease the adjustment.

### Sleep Disturbances

Sleep disturbances have been reported to have a physiological component. Infants born to women who drank heavily throughout pregnancy experience more interruptions in quiet sleep and are less apt to develop night-day sleep cycles than are other children (Sander et al. 1977). The sleep disturbances are believed to persist well into childhood, and children suffering from them may be helped with consistent bedtime routines, the use of “white noise” in a bedroom, snug bedclothes, and sensory integration therapy.

### Eating Problems

Infants with FAS may have eating problems because of neuromuscular delays, such as differences in the suck and swallow reflexes, or anatomical abnormalities, such as malformations of the oral cavity. Older children may eat little and gain weight slowly. Hypersensitivity to the feeling of food or utensils in their mouths can cause children to play with food in their fingers or to chew over and over without swallowing. Parents can ease these problems by allowing children ample time to eat, having reasonable expectations on portion size, and carefully controlling temperature and texture of foods. Reducing distractions, particularly for infants, also may improve their food intake.

## Improving Society’s Responses to FAS

Providing intervention early in a child’s life capitalizes on the brain’s plasticity and capacity for adaptation. However, the ability to provide early intervention for any developmental problem depends on at least two premises: that diagnosis is made efficiently and accurately, and that the nature of the disability is well enough understood so that targeted, effective interventions can be designed. Neither of these ideas has been fully researched within the field of FAS. The scope of the problems is far reaching, needing a response from the medical system, parents, the educational and legal systems, and the community at large.

### Medical System

The early diagnosis of FAS is the important first step in linking children with appropriate intervention strategies during the most formative years, but the rate of diagnosis varies with the individual physician and within some populations such as Native Americans that are considered “at risk.” A survey of pediatricians in Massachusetts found that although knowledge of FAS is high among members of this group, they feel unprepared to deal with the issues (Morse et al. 1992). In fact, more of the pediatricians had suspected FAS than had made the diagnosis. In some settings, physicians are reluctant to give a diagnosis that they see as little more than a label that does not benefit the child and may be derogatory. In other settings, the diagnosis is overused, with the developmental problems of most children being attributed to prenatal alcohol exposure whether or not that was the case (J. Nanson, J. Aase, K. Jones, personal communication, April 1993).

Physicians who better understand FAS can form effective partnerships with parents (Olsen et al. 1992). Together, they can develop realistic expectations for the FAS child’s growth and development. In addition, physicians who are working closely with the biological parents of children with FAS have an opportunity to identify a parent’s ongoing substance abuse and to make referrals for substance abuse treatment.

### Parents

Biological, foster, and adoptive parents of children with FAS report inadequate access to information about FAS, difficulties obtaining a diagnosis of FAS, and sparse intervention resources for their children. They express concern over the behaviors, cognitive skills, and physical development of their children. In the absence of information and professional support, they may become frustrated and blame their children’s problems on themselves. The difficulties faced by parents of children with FAS—with the children themselves and with finding adequate support services—often cause rapid turnover in foster placements and contribute to failed adoptions.

Teaching parents ways to help children reach their potential is an effective therapeutic strategy; it provides parents with specific techniques while capitalizing on their knowledge of their children’s unique strengths and needs. Support groups further empower parents by giving them the knowledge they need about FAS. Informed parents also are better able to distinguish the physical or neurological bases of the behavioral and learning problems. Knowledge about FAS allows parents to realistically assess their children’s behavioral and learning abilities and to help teachers, health care providers, and other caregivers understand and provide for their children’s needs.

### Educational System

The children with FAS may be inappropriately placed within the school system if their condition has not been accurately diagnosed or if there is a misunderstanding of FAS by the school system. The learning and behavior problems of children with FAS are often inconsistent, thereby masking their educational needs and making it difficult to assess whether they require special classes. Some of these children are incorrectly considered to be emotionally disturbed, and children who could potentially function in regular classrooms with support may instead be placed in special education classes.

Many school systems base eligibility for special education on IQ scores, eliminating children with IQ’s above 69 even if they exhibit CNS problems, such as hyperactivity, sensory hypersensitivity, or learning disabilities. Neurobehavioral issues, such as aggression or temper tantrums, can cause some children with FAS to be labeled as bad or malicious, compromising appropriate intervention. In addition, teachers lack guidelines and protocols to address the unique problems of children with FAS.

### Legal System

#### Custody

Children with FAS are often placed within the foster/adoptive care system. Placing children is more difficult when their behaviors are not clearly understood and no definite treatment protocols are available. Families who assume temporary custody of children with FAS often become frustrated with their lack of knowledge and support and terminate their responsibility for the children.

#### Arrests

No guidelines exist for dealing with adolescents with FAS who have extreme behavioral problems. When these adolescents are arrested for their behavior, judges may make court decisions based on the assumption that adolescents with FAS understand the consequences of their actions, which they often do not. The behaviors that brought the adolescents to the court are not seen as a function of their disability. To prevent adolescents with FAS from repeating their antisocial behaviors, disciplinary programs should be based on an understanding of the neuropsychological and learning problems associated with FAS. These problems include difficulties in processing information, generalizing knowledge from one situation to another, and understanding cause and effect relationships.

### Community

Because the problems encountered by children with FAS and their families are multifaceted, there is a need for coordinated intervention approaches. Comprehensive care incorporating and coordinating medical, psychological, educational, and social problems will help maximize each child’s development in a more cost-effective manner. Such cooperation also will eliminate redundancies of treatment and allow problems to be recognized in early, more treatable stages.

The disabilities of FAS make affected children more vulnerable to the adverse effects of unstable or inconsistent environments. The complex interrelationships of environmental stress, inadequate parenting, lack of enrichment, and poor nutrition all contribute to these children’s poor development (Rosett and Weiner 1984). Sobriety by both parents is essential to the child’s optimal development, because parents who are actively drinking are unable to deal effectively with their child. Therefore, community health policy must include the provision of treatment for parents and reduce existing barriers to treatment for women, such as lack of child care or an unwillingness to treat women during their pregnancies.

## Focusing FAS Research

Over the past 20 years, only a small fraction of research on FAS has focused directly on the affected children. The research has concentrated on finding the mechanisms of alcohol’s damage, proving alcohol causes damage, and demonstrating that treating women during pregnancy can prevent FAS. This basic research has built a foundation of understanding that can be used to help design systematic interventions for the children themselves. A better understanding of the children with FAS needs to be obtained through the following methods:

- Conducting neuropsychological evaluations to determine how FAS is similar to and different from other CNS disorders
- Improving the accurate diagnosis of FAS, including developing specific protocols for pediatricians and educating pediatricians on the value of making a diagnosis of FAS
- Looking to existing understanding of developmental disabilities and learning disabilities for clues to successful models that can be tested with FAS
- Distinguishing FAS from the effects of fetal cocaine or other illicit drug exposure, and clarifying the ways in which the disabilities and interventions for these conditions are different
- Moving away from an exclusively mental retardation model that is not representative of all children with FAS.

Although the research is far from complete, the common characteristics seen in children with FAS provide invaluable information to caregivers and point to areas for future research. As the understanding of FAS increases, more effective interventions for children at home and school and in the community will be developed, and medical, social service, and educational professionals will be better equipped to address the complex problems that face affected children and their families.

## Footnotes

1 Average intelligence ranges from 90 to 110. A person with an IQ below 70 is considered mentally retarded.

2 A pilot program is a small study that is less sophisticated and rigorous than a traditional scientific study. The pilot study is used to test and refine the methods that can then be applied to a larger study.

3 Although the report does not provide a description of the intervention, early intervention generally consists of evaluation and therapy to address developmental problems in cognition, motor skills, speech and language, and social skills.
